# ﻿The species of the genus *Zaitzevia* Champion, 1923 (Coleoptera, Elmidae) from Shaanxi Province, China

**DOI:** 10.3897/zookeys.1264.156144

**Published:** 2025-12-16

**Authors:** Dong-Ju Bian, Manfred A. Jäch

**Affiliations:** 1 CAS Key Laboratory of Forest Ecology and Silviculture, Institute of Applied Ecology, Chinese Academy of Sciences, Shenyang, 110016, China Institute of Applied Ecology, Chinese Academy of Sciences Shenyang China; 2 Natural History Museum Vienna, Burgring 7, 1010 Wien, Austria Natural History Museum Vienna Wien Austria

**Keywords:** Key, Macronychini, new species, riffle beetles, taxonomy, *
Zaitzeviaria
*

## Abstract

A synopsis of the species of the genus *Zaitzevia* Champion, 1923 from Shaanxi Province (China) is provided. Five new species are described: *Z. coronifer***sp. nov.**, *Z. disparilis***sp. nov.**, *Z. hybrida***sp. nov.**, *Z. pilosa***sp. nov.**, and *Z. robusta***sp. nov.** These five new species are not confined to Shaanxi but recorded also from other provinces: *Z. coronifer* (Hubei), *Z. disparilis* (Anhui, Gansu, Guizhou, Hunan), *Z. hybrida* (Gansu, Sichuan, Yunnan), *Z. pilosa* (Hubei, Hunan), and *Z. robusta* (Hubei, Sichuan). A key to the six *Zaitzevia* species currently known from Shaanxi, as well as photographs of the habitus and the aedeagi, are provided. The morphological delimitation of the genera *Zaitzevia* and *Zaitzeviaria* Nomura, 1959 is briefly discussed on the basis of the newly established *Zaitzevia hybrida* species group, which is wide-spread in the Himalaya and China and combines diagnostic characters of *Zaitzevia* as well as *Zaitzeviaria*. In addition, an updated checklist of the 21 species of *Zaitzevia* now recorded from China is presented. The genus *Zaitzevia* is recorded for the first time from Anhui, Gansu, and Guizhou. *Zaitzevia fengtongzhaiensis* Jiang & Chen, 2023 is recorded for the first time from Gansu and Yunnan. Based on particular morphological characters, the specimens recorded by Bian and Zhang (2022) from Shaanxi and Yunnan as *Z. chenzhitengi* are here regarded as a closely related, undescribed species.

## ﻿Introduction

The genus *Zaitzevia* Champion, 1923 includes 30 valid species distributed in Asia and North America, including Mexico (Jäch et al. 2016; Jiang and Wang 2020, 2021; Bian and Zhang 2022; Iwata et al. 2022; Jiang and Chen 2023; Bian and Hu 2024; Peng et al. 2024). Sixteen species of *Zaitzevia* are currently known from China. One of these, *Z. chenzhitengi* Jiang & Wang, 2020, has been recorded from Shaanxi Province by Bian and Zhang (2022). In the course of the China Water Beetle Survey (CWBS) five new species of *Zaitzevia* were collected from 12 localities in Shaanxi in June 1998. In the present paper we describe these five new taxa, raising the total number of *Zaitzevia* species of this province to six. In addition, we present an updated checklist of the *Zaitzevia* species from China and a key to the species from Shaanxi.

## ﻿Materials and methods

Most of the specimens treated herein were collected during the China Water Beetle Survey (CWBS) between 1993 and 2004 (for additional locality information and habitat photographs see Jäch and Ji 1995, 1998, 2003). The unpublished locality descriptions of the specimens collected in Hubei in October 2004 are listed below:

CWBS loc. 522: Hubei Province, Shennongjia Forest District; ca 10 km E of Muyu, Chaiqi Village; stream, ca 2–4 m wide, large boulders and gravel, flowing through steep gorge with bushes, ca 1,600 m a.s.l., geology: limestone, sandstone; 9.X.2004; leg. H. Schönmann & M. Wang.

CWBS loc. 523: Hubei Province, Shennongjia Forest District; ca 3 km N of Muyu, Duanjiang Village; stream (right tributary of River Xiangxi), 3–4 m wide, rocks and gravel, shaded by bushes, ca 1,300 m a.s.l., geology: sandstone; 10.X.2004; leg. H. Schönmann & M. Wang.

CWBS loc. 525: Hubei Province, Shennongjia Forest District; ca 5 km SW of Muyu; stream (right tributary of River Xiangxi), < 2 m wide, gravel, flowing through steep rocky gorge with bushes, ca 1,350 m a.s.l., geology: sandstone; 10.X.2004; leg. H. Schönmann & M. Wang.

CWBS loc. 527: Hubei Province, Shennongjia Forest District; ca 4 km NW of Muyu, margin of Shennongjia Nature Reserve; cold stream, 3–5 m wide, large sandstone boulders, ca 1,500 m a.s.l., 11.X.2004; leg. H. Schönmann & M. Wang.

CWBS loc. 528: Hubei Province, Shennongjia Forest District; ca 10 km E of Muyu, Chaiqi Village, 1 km N of CWBS loc. 522; stream, ca 1–2 m wide, sandstone gravel and large boulders, flowing between bushes, ca 1,700 m a.s.l., 12.X.2004; leg. H. Schönmann & M. Wang.

CWBS loc. 529: Hubei Province, Shennongjia Forest District; ca 20 km E of Chaiqi Village; mountain stream, ca 3–5 m wide, flowing through forest (*Larix*, *Abies*, *Betula*), with large boulders, coarse gravel, fallen trees, ca 2,150 m a.s.l., 12.X.2004; leg. H. Schönmann & M. Wang.

CWBS loc. 531: Hubei Province: Shennongjia Forest District; ca 5 km E of Muyu, Tong Mu Village; stream, ca 2–3 m wide, slowly flowing between agricultural fields, ca 1,250 m a.s.l., geology: sandstone, marble; 13.X.2004; leg. H. Schönmann & M. Wang.

CWBS loc. 536: Hubei Province, Shennongjia Forest District; ca 25 km W of Muyu–Shennongjia Pass; stream, ca 3–5 m wide, on densely forested mountain, limestone rock, gravel, ca 1.600 m a.s.l.; 15.X.2004; leg. H. Schönmann & M. Wang.

CWBS loc. 539: Hubei Province: Shennongjia Forest District; ca 35 km N of Muyu, along road to Fang Xian; stream (tributary of Nan He), ca 2–3 m wide, flowing between abandoned fields and forest, ca 1,600 m a.s.l., geology: sandstone and limestone; 17.X.2004; leg. H. Schönmann & M. Wang.

CWBS loc. 540: Hubei Province: Shennongjia Forest District; ca 35 km N of Muyu, along road to Fang Xian; thermal spring (tributary of Nan He), ca 0.5 m wide, opposite of CWBS 539, ca 1,600 m a.s.l.; 17.X.2004; leg. H. Schönmann & M. Wang.

CWBS loc. 543: Hubei Province, Enshi Autonomous Prefecture; Tiechanghuang Forest Park, ca 60 km SSW of Badong; cold stream, ca 0.5–1.0 m wide, *Pinus*/*Cunninghamia* forest, gravel, trapped plant debris, submerged vegetation, ca 1,600 m a.s.l.; 19.X.2004; leg. H. Schönmann & M. Wang.

CWBS loc. 549: Hubei Province, Enshi Autonomous Prefecture, ca 50 km W of Enshi, along road to Lichuan; short spring, ca 0.2–1.0 wide, on karst plateau, some submerged vegetation, ca 1,600 m a.s.l.; 23.X.2004; leg. H. Schönmann & M. Wang.

This study is based on 1,383 specimens collected between 1991 and 2019 in the provinces of Anhui, Gansu, Guizhou, Hubei, Hunan, Shaanxi, Sichuan, and Yunnan. The holotypes of the new species are deposited in the Institute of Applied Ecology, Chinese Academy of Sciences, Shenyang, China (**IAECAS**), paratypes are housed in the IAECAS, the Natural History Museum Vienna, Austria (**NMW**), coll. David Boukal, České Budějovice, Czechia (**CBC)** and coll. Andreas Pütz, Eisenhüttenstadt, Germany (**CPE**).

Specimens were examined with a Leica M205c stereomicroscope and an Olympus BX51 compound microscope. Male genitalia were placed in concentrated lactic acid in a cavity slide for at least several hours before they were examined. Habitus and genitalia photographs were made with KEYENCE VHX-2000 – Super Resolution Digital Microscope System. The first strial interval refers to the sutural interval. Label data are cited verbatim, with separate lines on the same label indicated by a backslash “\”; different labels are separated by a vertical line “|”. Measurements: **BL**—body length (= PL+EL), **BW**—maximum width of body (= EW), **EL**—elytral length, **EW**—maximum width of elytra, **PL**—pronotal length, **PW**—maximum width of pronotum.

## ﻿Taxonomy

### ﻿Family Elmidae Curtis, 1830

**Elmidae Curtis, 1830: 249.**

#### ﻿ Macronychini

Taxon classificationAnimaliaColeopteraElmidae

Gistel, 1848

B199F4E7-403A-5378-8713-00271D48F54C

Macronychini Gistel, 1848: unnumbered page between columns 400 and 409. Type genus: Macronychus Müller, 1806.

##### Notes.

No modern classification of Elmidae has been published so far. *Zaitzevia* and several other closely related genera are currently placed in the tribe Macronychini. But due to the lack of comprehensive phylogenetic studies, it is currently not possible to provide a valid subfamily classification.

#### ﻿Genus Zaitzevia

Taxon classificationAnimaliaColeopteraElmidae

Champion, 1923

D38B58A7-7206-5B23-9293-8BD4E30D1EAC

Zaitzevia Champion, 1923: 170. Type species: Zaitzevia solidicornis Champion, 1923. Awadoronus Kôno, 1934: 127 (synonymized by Nomura 1958). Type species: Awadoronus awanus Kôno, 1934.

#### ﻿ Zaitzevia robusta sp. nov.

Taxon classificationAnimaliaColeopteraElmidae

74FFDBBB-BD76-5F82-ABB5-B71AF1F41256

https://zoobank.org/B34A91B3-C625-479E-B681-BD226273989D

**Figure 1.**
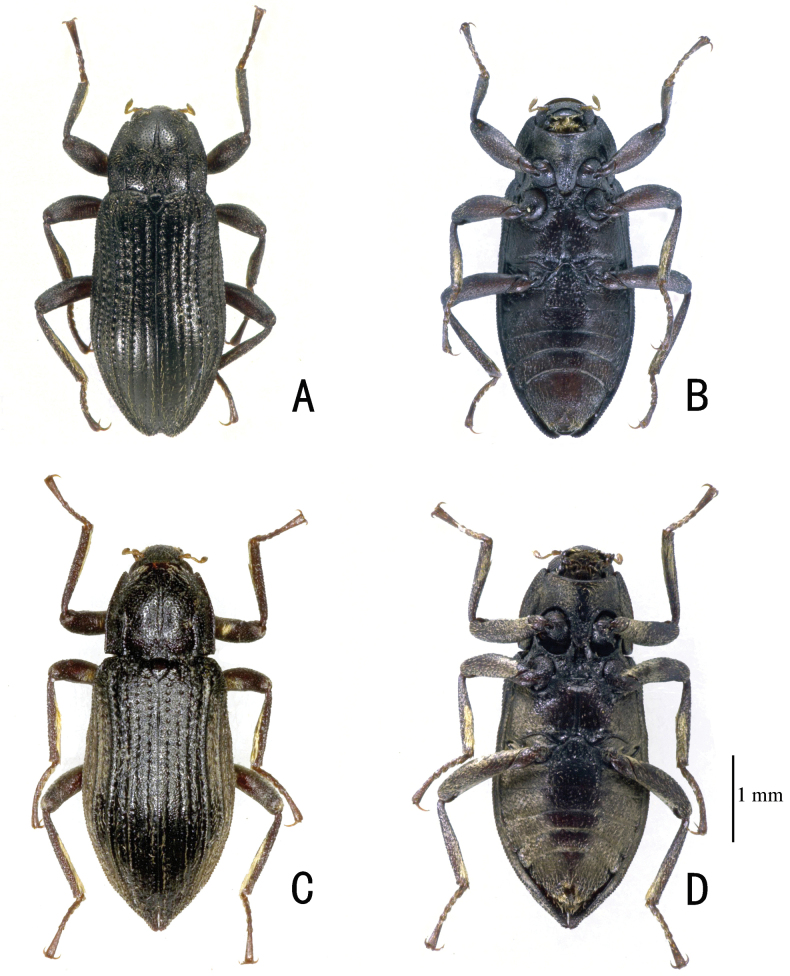
Habitus. *Zaitzevia robusta* sp. nov. **A, B.** Holotype, male; **C, D.** Paratype, female. **A, C.** Dorsal view; **B, D.** Ventral view.

Figs 1A–D, 5A–C, 10A–C

##### Material examined.

(41 exs) ***Holotype***: China • ♂ (IAECAS): “China: Shaanxi, \ Foping County \ Gaozhuanggou | 33°34′18″N, 107°57′56″E \ 1035 m, 2019. VI. 21 \ Leg. Tong”. ***Paratypes***: China, Shaanxi • 1 ♂, 5 ♀♀ (IAECAS): “China: Shaanxi \ Qinling, NingShan County, \ Ningdong Forest Agency | Dacigou \ 2005.5.11 \ 1398 m \ Leg. Wang” • 5 ♀♀ (IAECAS): “China: Shaanxi \ Qinling, Ningshan County \ Ningdong Forest Agency | Dacigou, 2005.VI.11 \ 1437 m, Leg. Wangm [Wang Miao]” • 2 ♂♂ (IAECAS): “China –Shaanxi \ Zhouzhi County l.w [leg. Wang] | Houzhenzi Ca1200m \ 2.VI 1998” [CWBS 308] • 2 ♀♀ (NMW): “CHINA: Shaanxi, 2.6.1998 \ Zhouzhi County, ca. 1200m \ 2km W Houzhenzi Nat. Res. \ leg. M. Wang (CWBS 308)” • 1 ♀ (NMW): “China: Shaanxi, 3.6.1998 \ Houzhenzi County, ca. 1300 m \ 2km E Houzhenzi Nat. Res. \ leg. M. Wang (CWBS 309)” • 1 ♂, 2 ♀♀ (NMW): “China: Shaanxi, 9.6.1998 \ Tiantai Shan Forest Park \ Feng Co., 7km NE Qinling \ Train Station, ca. 2000m \ leg. M. Wang (CWBS 319)” • 1 ♂ (IAECAS): “China –Shaanxi \ Feng County I. [leg.] W [Wang] | Qinling Train Station \ ca. 1900 m; 9.VI.1998” [CWBS 320] • 1 ♂ (NMW): “China: Shaanxi, 10.6.1998 \ Tiantai Shan Forest Park \ Feng Co., 7km NE Qinling \ Train Station, ca. 1850m \ leg. M. Wang (CWBS 321)” • 2 ♂♂, 5 ♀♀ (NMW), 1 ♂, 1 ♀ (CPE): “China: Shaanxi, Qin Ling Shan \ 110.06E, 34.27N \ Hua Shan Mt. N Valley, 1200–1400 m \ 180 km E Xian, sifted [crossed out with black pen] 18. /20.08. 1995, leg. A.Pütz” – type locality of *Elmomorphus catenatus* Selnekovič, Jäch & Kodada, 2024 (Dryopidae); Sichuan • 1 ♂ (IAECAS): “China: Sichuan \ Aba Pref., Mao County \ Daguan Town | Dadian Vill. 2012.04 [day not mentioned on label] \ 31°56'7.7"N, 103°40'18.8"E \ ca. 1830 m, leg. Wang & Guo” • 1 ♂, 3 ♀♀ (NMW): “China: Sichuan, 29.7.1998 \ Mao Xian Co., Jiuding Shan \ 7 km NE Mao Xian, ca. 1850m \ Schönmann, Ji, Wang (CWBS 335)” • 1 ♂ (IAECAS): “China-Sichuan \ Mao cty Jiudingshan | 1950m leg Ji & W [Wang] \ 7.29.1998” [CWBS 336] • 1 ♂, 1 ♀ (NMW): “China: Sichuan, 29.7.1998 \ Mao Xian Co., Jiuding Shan \ 10 km NE Mao Xian, ca. 1950m \ Schönmann, Ji, Wang (CWBS 336)” • 1 ♂ (NMW): “China: Sichuan, 30.7.1998 \ Mao Xian Cty., Jiuding Shan \ 6 km NE Mao Xian, ca. 1750m \ Schönmann, Ji, Wang (CWBS 340)”; Hubei • 1 ♂ (NMW): “China: Hubei, 19.10.2004 \ Tienchanghuang Forest Park \ Enshi [Pref.], 60 km SSW Badong \ 1 600 m, leg. Schönmann \ & Wang (CWBS 543)”.

**Table 1.** Checklist of the Chinese species of *Zaitzevia*.

*Z. acuta* Bian & Hu, 2024	Guangdong
*Z. babai* Nomura, 1963	Taiwan
*Z. chenzhitengi* Jiang & Wang, 2020	Sichuan, ? Shaanxi, ? Yunnan
*Z. coronifer* sp. nov.	Hubei, Shaanxi
*Z. disparilis* sp. nov.	Anhui, Gansu, Guizhou, Hunan, Shaanxi
*Z. fengtongzhaiensis* Jiang & Chen, 2023^1^	Gansu, Sichuan, Yunnan
*Z. formosana* Nomura, 1963	Taiwan
*Z. gaoligongensis* Bian & Zhang, 2022	Yunnan
*Z. hybrida* sp. nov.	Gansu, Shaanxi, Sichuan, Yunnan
*Z. muchenae* Bian & Zhang, 2022	Yunnan
*Z. nanlingensis* Bian & Hu, 2024	Guangdong
*Z. parallela* Nomura, 1963	Taiwan
*Z. pilosa* sp. nov.	Hubei, Hunan, Shaanxi
*Z. reniformis* Bian & Zhang, 2022	Yunnan
*Z. robusta* sp. nov.	Hubei, Shaanxi, Sichuan
*Z. sichuanensis* Jiang & Chen, 2023	Sichuan
*Z. tangliangi* Jiang & Wang, 2021	Hubei
*Z. triangularis* Peng, Bian & Wang, 2024	Shanxi
*Z. tsushimana* Nomura, 1963	Jilin; Japan; Korea; Russia
*Z. xiongzichuni* Jiang & Wang, 2020	Yunnan
*Z. yingzuijieensis* Jiang & Chen, 2023	Hunan

**^1^***Zaitzevia fengtongzhaiensis* is obviously wide-spread in China. We have examined specimens from three provinces, deposited in the NMW and CPE: Gansu (first record, 1 ♂, CWBS 322), Sichuan (37 exs, CWBS 227, 332, 337–339; 3 exs, Ganzi Pref., Gongga Shan, 29.36°N, 102.04°E, 2,100 m a.s.l., 28./31.V.1997, leg. E. Pütz; 3 exs, Qingchengshan, 65 km NW Chengdu, 10 km W Taiping, ca 600 m a.s.l., 4.V.1997, leg. E. Pütz), and Yunnan (first record, 1 ♂, 2 ♀♀, CWBS 56). We have examined one male from Hubei (CWBS 522), which agrees in most principal characters with the specimens from Gansu, Sichuan, and Yunnan, but its endophallic teeth are larger and straighter, and the apex of the penis is slightly wider. Probably, this specimen represents a closely related, undescribed species. More material and molecular data are needed to verify this assumption.

##### Differential diagnosis.

*Zaitzevia robusta* sp. nov. is the largest species (BL 3.14–3.80 mm) of the genus described so far. The new species resembles *Z. chenzhitengi*, *Z. fengtongzhaiensis*, *Z. muchenae*, and *Z. sichuanensis*. Males can be distinguished from *Z. chenzhitengi* and *Z. fengtongzhaiensis* mainly by the longer aedeagus, which is distally not acuminate but apically rounded in ventral/dorsal views, and the elytral apices are more acuminate in *Z. fengtongzhaiensis* and the holotype of *Z. chenzhitengi*; males are distinguished from *Z. muchenae* by the more robust body, the longer and broader pronotal median sulcus and by the distinctly longer and broadly rounded aedeagus; males differ from *Z. sichuanensis* in the larger and more robust body, and the straight and longer, apically broadly rounded aedeagus; from *Z. triangularis* (BL: 2.9–3.2 mm) males differ in the slightly larger body size, the less acuminate elytral apices, and in the broadly rounded, not triangular aedeagal apex.

##### Description.

**Male** (holotype). BL 3.6 mm, BW 1.5 mm. Body elongate obovate. Antennae yellowish brown, dorsum black, ventral surface reddish brown to dark brown, and legs reddish brown.

Labrum smooth and shiny, with only a few punctures and scarce pubescence, lateral margins densely covered with long setae; anterior margin broadly rounded, not emarginate. Clypeus and frons densely punctate and pubescent, sparsely granulate.

Pronotum (PL 0.9 mm, PW 1.1 mm) broadest at basal 0.3, slightly narrowed posteriorly and distinctly attenuate anteriorly. Disc in basal 0.3 rugose, sparsely punctate and pubescent, in distal 0.7 smooth and shiny, densely punctate and sparsely pubescent. Lateral areas densely granulate and sparsely pubescent. Anterior corners acute, slightly produced, posterior corners almost right-angled. Lateral margin narrowly rimmed. Median sulcus extending from basal 0.2–0.8, broadest at basal 0.3, gradually narrowed anteriorly and posteriorly. Sublateral carinae present in basal 0.4 bending outwards at basal 0.3.

Elytra (EL 2.7 mm, EW 1.5 mm) broadest at distal 0.33, slightly narrowed anteriorly and distinctly attenuate posteriorly. Lateral margin serrate. Strial punctures small in basal 0.5, separated by 0.5 their diameters, punctures in distal 0.5 smaller and well separated (1–3 × their diameters). Intervals rugose, sparsely punctate and pubescent. Intervals 2–4 slightly elevated in basal 0.2. Intervals 5, 7 and 8 carinate. Elytral apices separately rounded, densely granulate.

Prosternum with disc rugose, almost glabrous; lateral parts of prosternum granulate and pubescent. Prosternal process gradually narrowed from base to broadly rounded apex; disc glabrous, surface coarse, with a few small setae and scattered granules; lateral margins distinctly rimmed. Metaventrite broadly impressed medially, disc smooth and shiny, with some longitudinal rugosities; median sulcus broad in posterior 0.8 and narrow in anterior 0.2; lateral areas densely pubescent and sparsely granulate; with a row of large punctures behind of mesocoxa and another row of small punctures in front of metacoxa; base with a pair of pits on each side of median sulcus.

Ventrite I with a pair of admedian carinae; area between these carinae (disc) flat or slightly concave, anteriorly rugose, remaining parts with small punctures, interstices micropunctate. Lateral areas of ventrites I–V densely pubescent and sparsely granulate. Middle of ventrites II–IV and basal 0.5 of ventrite V glabrous and sparsely punctate. Distal 0.5 of ventrite V densely granulate; lateral margins sometimes fringed with very short, inconspicuous spines; apex deeply emarginate medially, lateral corners produced, tooth-like and slightly bent ventrad.

Mesal margin of mesotibia slightly expanded around middle.

**Aedeagus**. 1.9 mm long, elongate, penis ~ 2.2 × as long as phallobase. Lateral margins of penis in ventral/dorsal view more or less subparallel except for a very inconspicuous swelling and/or a very shallow emargination at apical 0.3; dorsal wide with a conspicuous gibbosity of variable size at apical 0.25 (best seen in lateral view); apex of penis broadly and evenly rounded in ventral/dorsal view, distinctly curved dorsad in lateral view. Endophallus in distal 0.33 with a pair of small curved teeth. Parameres short and inconspicuous, clinging to penis.

**Females**. Elytral apices not separately rounded but distinctly acuminate and produced to a remarkably variable extent; they vary from short to long and beak-like, and sometimes they are asymmetrically produced, being longer on one side than on the other. Metaventrite flat or slightly depressed, distinctly less strongly impressed than in males, smoother. Disc of ventrite I more or less convex. Ventrite V less widely and less deeply excised apically, lateral apical teeth shorter and smaller than in males; apical 0.33 of ventrite V less strongly granulate. The femora often appear to be more strongly-built in the males, but this depends largely on the body size (in larger specimens the femora are proportionately thicker).

##### Measurements.

Males: BL 3.50–3.80 mm (*n* = 14), BW 1.40–1.55 mm (*n* = 5); females: BL 3.14–3.78 mm (*n* = 25), BW 1.40–1.60 mm (*n* = 11).

##### Distribution.

Hubei, Shaanxi, Sichuan.

##### Etymology.

The name *robustus*, a Latin adjective, refers to the massive body.

#### ﻿ Zaitzevia pilosa sp. nov.

Taxon classificationAnimaliaColeopteraElmidae

0661EB0E-5132-5538-91AA-FD57374E301B

https://zoobank.org/2D6C8336-5CB4-4403-8521-E553D1BCE4C0

**Figure 2.**
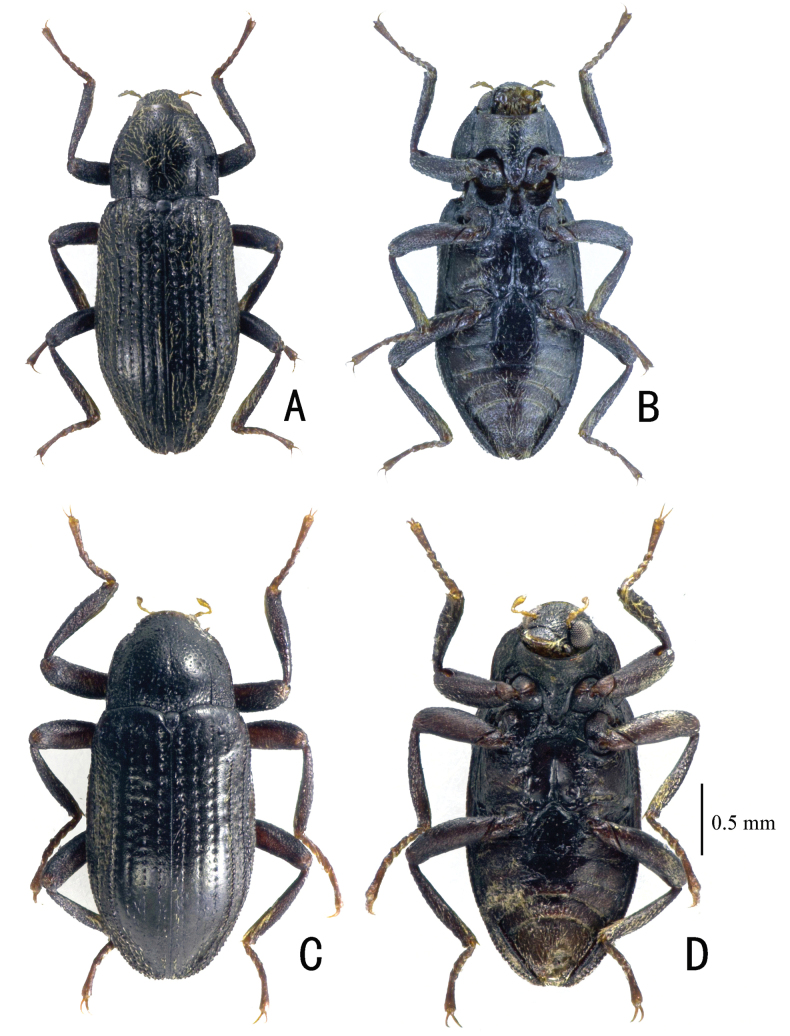
Habitus. **A, B.***Zaitzevia pilosa* sp. nov.; holotype, male; **C, D.***Z. coronifer* sp. nov., holotype, male; **A, C.** Dorsal view; **B, D.** Ventral view.

Figs 2A, B, 6A–C, 11A–C

Zaitzevia sp.: Jäch and Boukal 1995: figs 36, 37.

##### Material examined.

(237 exs) ***Holotype***: China • ♂ (IAECAS): “China: Shaanxi \ Qinling, Ningshan County \ Ningdong Forest Agency | Dacigou, 2005.VI.11 \ 1437 m, Leg. Wangm [Wang Miao]”. ***Paratypes***: China, Shaanxi • 3 ♀♀ (IAECAS), the same data as holotype • 1 ♀ (IAECAS): “China: Shaanxi \ Qinling, NingShan County, \ Ningdong Forest Agency | Dachigou \ 2005.5.11 \ 1398 m \ Leg. Bian” • 3 ♂♂, 1 ♀ (IAECAS): “China: Shaanxi, Ankang City, \ Ningshan County, Xunyang Dam | 33°33.473′N,108°32.808′E, \ 1355 m, 2019.8.20 \ Leg. Tong Y.F” • 2 ♂♂, 3 ♀♀ (NMW): “China: Shaanxi, 5.6.1998 \ Ningshan Co., ca. 1900m \ 5km NW Huoditang \ leg. M. Wang (CWBS 313)” • 18 exs (NMW): “China: Shaanxi, 5.6.1998 \ Ningshan Co., ca. 1650m \ 7km NW Huoditang \ leg. M. Wang (CWBS 314)” • 27 exs (NMW): “China: Shaanxi, 6.6.1998 \ Ningshan Co., ca. 1 500m \ 10 km NE Xunyangba \ leg. M. Wang (CWBS 315)”; Hubei • 11 exs (IAECAS): “China: Hubei \ Shennongjia For. Dist. | 1600 m, 2004.10.9 \ Leg. Wang (CWBS 522)” • 10 exs (NMW): “China: Hubei, 9.10.2004 \ Shennongjia Forest Distr. \ 10 km E Muyu, Chaiqi \ 1600 m, leg. Schönmann, \ & Wang (CWBS 522)” • 4 exs (NMW): “China: Hubei, 10.10.2004 \ Shennongjia Forest Distr. \ 3 km N Muyu, Duanjiang \ 1300 m, leg. Schönmann \ & Wang (CWBS 523)” • 3 exs (NMW): “China: Hubei, 10.10.2004 \ Shennongjia Forest Distr. \ 5 km SW Muyu \ 1350 m, leg. Schönmann \ & Wang (CWBS 525)” • 4 ♂♂, 4 ♀♀ (IAECAS): “China: Hubei \ Shennongjia Forest Dist. | 2004.10.11 \ Leg. Wang (CWBS 527)” • 7 exs (NMW): “China, Hubei, 11.10.2004 \ Shennongjia Forest Distr. \ 4 km NW Muyu \ 1800 m, leg. Schönmann \ & Wang (CWBS 527)” • 5 exs (NMW): “China: Hubei, 11.10.2004 \ Shennongjia Forest Distr. \ 3 km N Muyu, Chaiqi env. \ 1 700 m, leg. Schönmann \ & Wang (CWBS 528)” • 2 exs (NMW): “China: Hubei, 12.10.2004 \ Shennongjia Forest Distr. \ 20 km E Muyu, 10 km E \ Chaiqi, 2150 m, leg. Wang \ & Schönmann (CWBS 529)” • 4 exs (NMW): “China: Hubei, 15.10.2004 \ Shennongjia Forest Distr. \ 25 km W pass Muyu - \ Shennongjia \ 1650 m, leg. Schönmann \ & Wang (CWBS 536)” • 2 exs (NMW): “China: Hubei, 17.X.2004 \ Shennongjia Forest Distr. \ 35 km N Muyu \ 1600 m, leg. Schönmann \ & Wang (CWBS 539)” • 23 exs (IAECAS): “China: Hubei, Enshi \ 35 km N Muyu | 1600 m, 2004.10.17 \ Leg. Wang (CWBS 540)” • 5 ♂♂, 48 exs (IAECAS): “China: Hubei, Enshi \ Lichuan | 1600 m, 2004.10.23 \ Leg. Wang (CWBS 549)” • 33 exs (NMW): “China: Hubei, 23.10.2004 \ 50 km W Enshi \ 1 600 m, leg. Schönmann \ & Wang (CWBS 549)”; Hunan • 10 exs (NMW): “China, NW-Hunan 1993 \ Wulingyuan, N Dayong \ Zangjiajie [Zhangjiajie], 29.10., 650m \ leg. Schillhammer (1) [CWBS 20]” • 12 exs (NMW): same locality data, but “leg. Schönmann” • 1 ♂, 1 ♀ (IAECAS): “China: Hunan, Zhangjiajie \ Suoxiyu Nat. Res. | 650 m, 1993.10.29 \ Leg. L.Ji (CWBS 21)” • 1 ♂, 1 ♀ (NMW): “China, NW-Hunan 1993 \ Wulingyuan, N Dayong \ Zangjiajie [Zhangjiajie], 29.10., 650m \ leg. L. Ji (2) [CWBS 21]”.

##### Differential diagnosis.

This species is well characterized by its size (BL 2.4–2.9 mm) in combination with the conspicuous dorsal pilosity (if not rubbed off, as in many older specimens) and the granules on the elytral interval 5 more or less reaching the elytral base.

In habitus and body size the new species vaguely resembles *Z. yingzuijieensis*. It can be distinguished from the latter mainly by the conspicuous pubescence, the black dorsum (dark brown in *Z. yingzuijieensis*), the narrow median pronotal sulcus extending from basal 0.2–0.6 (< 0.33 length of pronotum in *Z. yingzuijieensis*), the distinct sublateral pronotal carinae (faint in *Z. yingzuijieensis*), the apex of the prosternal process being narrowly rounded (broadly rounded in *Z. yingzuijieensis*), the symmetrical aedeagus (asymmetrical in *Z. yingzuijieensis*), and several differences in the endophallus.

##### Description.

**Male** (holotype). BL 2.5 mm, BW 1.1 mm. Body slender. Dorsal surface black, ventral surface dark brown to black. Legs dark brown to black but tarsi reddish brown. Antennae yellowish brown.

Labrum wider than long, microreticulate in basal 0.33 but smooth and shiny in distal 0.67; punctures and pubescence moderately dense. Anterior margin arched, not emarginate, densely pubescent laterally. Clypeus and frons densely pubescent and granulate. Clypeal suture shallowly impressed.

Pronotum (PL 0.7 mm, PW 0.8 mm) subparallel in basal 0.4, distinctly attenuate anteriorly. Lateral margin narrowly rimmed, anterior 0.5 slightly serrate. Anterior corners acute, slightly produced; posterior corners almost right-angled. Disc smooth and shiny, densely punctate and covered by golden setae. Median sulcus extending from basal 0.2–0.6. Sublateral carinae present in basal 0.4. Areas near anterior and posterior corners densely and finely granulate.

Elytra (EL 1.8 mm, EW 1.1 mm) broadest at anterior 0.67, slightly narrowed anteriorly and distinctly attenuate posteriorly, apices separately rounded. Strial punctures moderately large in basal 0.5, separated by 1–2 × their diameters, distinctly smaller and well separated in distal 0.5. Intervals flat, sparsely punctate, each with a row of moderately long setae. Carinae on intervals 5, 7 and 8 complete.

Prosternum laterally densely pubescent; prosternal process gradually narrowed from base to basal 0.8, distinctly narrowed in distal 0.2, apex narrowly rounded; lateral margin distinctly rimmed; disc rugose, sparsely punctate and pubescent. Metaventrite broadly and shallowly impressed, disc smooth and shiny, sparsely punctate, and pubescent, somewhat rugose in posterior 0.5; median sulcus broad in posterior 0.8, and narrowed in anterior 0.2; with a row of large punctures behind of mesocoxae and a shallow groove in front of metacoxae; base with a pair of large pits on each side of median sulcus.

Ventrite I with a pair of admedian carinae; disc distinctly impressed, microreticulate and sparsely punctate. Middle of ventrites II–IV and anterior 0.25 of ventrite V smooth and shiny, sparsely punctate and sparsely pubescent. Lateral areas of ventrites I–V densely pubescent. Distal 0.75 of ventrite V densely granulate and sparsely pubescent. Lateral margins of ventrite V fringed with short spines; apex of ventrite V widely and deeply emarginate medially; lateral corners forming long teeth.

**Aedeagus.** 1.1 mm long, elongate, cylindrical. Penis ~ 2.5 × as long as phallobase, subparallel in basal 0.7, distinctly narrowed in distal 0.3, apex acute. Sclerotizations of endophallus somewhat resembling a pair of overlapping banana leaves; apex of endophallus with a pair of large curved double-pointed teeth. Parameres very slim, reaching apical 0.3 of penis.

A specimen from Hunan (CWBS 20) with everted endophallus has been illustrated by Jäch and Boukal (1995: figs 36, 37); unfortunately, this particular specimen could not be traced and is therefore not designated as paratype.

**Females**. Secondary sexual dimorphism rather poorly developed. Elytra apically often, but not always, more acuminate than in males. Metaventrite, including discrimen, a little less strongly or less comprehensively impressed. Disc of ventrite I less distinctly impressed. Ventrite V less widely and less deeply excised apically, lateral apical teeth distinctly shorter and smaller than in males; apical 0.33 of ventrite V less strongly granulate. Femora on average insignificantly slimmer than in males.

##### Measurements.

Males: BL 2.5–2.8 mm, BW 1.1–1.2 mm (*n* = 10); females: BL 2.4–2.9 mm, BW 1.1–1.2 mm (*n* = 12).

##### Variability.

Elytral apices of males usually conjointly rounded, but sometimes they are separately rounded or slightly excised. In some specimens from Hubei (e.g., CWBS 529, 549), the elytral intervals are tendentially more convex, and specimens from Hunan (CWBS 20) differ from the specimens from Shaanxi in the usually more glabrous surface, larger and deeper elytral punctures, and more convex elytral intervals, and the larger aedeagus (~ 1.30–1.36 mm long).

##### Distribution.

Hubei, Hunan, Shaanxi.

##### Habitat.

This species is normally found in cold mountains streams, but 23 specimens were surprisingly collected in a hydrothermal spring in Hubei (CWBS 540).

##### Etymology.

The epithet is derived from the Latin adjective *pilosus* (hairy, pilose) and refers to the pronounced pilosity on the dorsal body surface often observed in fresh specimens.

#### ﻿ Zaitzevia coronifer sp. nov.

Taxon classificationAnimaliaColeopteraElmidae

C97BBB86-9AEE-5623-AF73-446170D26E7A

https://zoobank.org/C9FC9D76-AEEC-4C3D-A29A-28B641CD4095

Figs 2C, D, 7A–C, 11D–F

##### Material examined.

(117 exs) ***Holotype***: China • ♂ (IAECAS): “China –Shaanxi \ Zhouzhi County l.w [leg. Wang] | Houzhenzi Ca1200m \ 2.VI 1998” [CWBS 308]. ***Paratypes***: China, Shaanxi • 5 ♀♀ (IAECAS), the same data as holotype • 35 exs (NMW): “China: Shaanxi, 2.6.1998 \ Zhouzhi County, ca. 1200m \ 2km W Houzhenzi Nat. Res. \ leg. M. Wang (CWBS 308)” • 2 ♂♂, 3 ♀♀ (IAECAS): “China: Shaanxi, Ankang City, \ Ningshan County, Xunyang Dam | 33°33.473′N,108°32.808′E, \ 1355 m, 2019.8.20 \ Leg. Tong Y.F” • 1 ♂, 1 ex. (IAECAS): “China-Shaanxi \ Mei County L [leg.]. W [Wang] | Tangyu ca. 1100 m \ 1. VI. 1998” [CWBS 307] • 1 ♂ (NMW): “China: Shaanxi, 4.6.1998 \ Foping County, ca. 1 300m \ 5km S Longcaoping \ leg. Wang (CWBS 312)” • 2 ♂♂, 8 ♀♀ (IAECAS): “China-Shaanxi \ Ningshan L. [leg.] W [Wang] | Xunyangba ca. 1500 m \ 6. VI. 1998” [CWBS 315] • 40 exs (NMW): “China: Shaanxi, 6.6.1998 \ Ningshan Co., ca. 1 500m \ 10 km NE Xunyangba \ leg. M. Wang (CWBS 315)” • 1 ♂, 1 ♀ (NMW): “China: Shaanxi, 8.6.1998 \ 2km SE Liuba, 1 400 m \ (Zhangliangmiao) \ leg. M. Wang (CWBS 317)” • 22 exs (CBC, NMW): “China, 17. – 22.VI. \ Shaanxi prov. 1991 \ Hua Shan peak env. \ 100 km E of Xi’an \ Z. Kejval lgt.”; Hubei • 3 exs (NMW): “China: Hubei, 13.10.2004 \ Shennongjia Forest Distr. \ 5 km E Muyu, Tong Mu \ 1 250 m, leg. Schönmann \ & Wang (CWBS 531)” • 2 ♂♂, 4 ♀♀ (IAECAS): “China: Hubei \ Shennongjia For. Dist. | 1600 m, 2004.10.15 \ Leg. Wang (CWBS 536)”.

##### Differential diagnosis.

In Shaanxi, this species is similar to *Z. pilosa* sp. nov. in body size (BL 2.3–2.7 mm) and habitus, but it can be easily distinguished from the latter by the less pronounced dorsal pilosity, the granules on the elytral interval 5 not reaching the elytral base and by the glabrous shoulders; the aedeagus differs mainly in the more strongly produced apex and the absence of endophallic teeth.

*Zaitzevia coronifer* also resembles *Z. tangliangi*, but it can be easily distinguished from the latter, e.g., by the stronger pronotal punctation, more distinct sublateral pronotal carinae, stronger tibiae (especially foretibiae), and by the clearly different aedeagus, being less elongate, apically more acuminate, with characteristic endophallic sclerotizations.

The new species is externally also somewhat similar to *Z. tsushimana*, which is, however, markedly smaller (BL 1.8–2.2 mm), with its median pronotal groove more or less reaching the pronotal base, and its elytral carinae being located on intervals 5–7 (see Jung et al. 2015; Iwata et al. 2022: fig. 3G).

**Figure 3.**
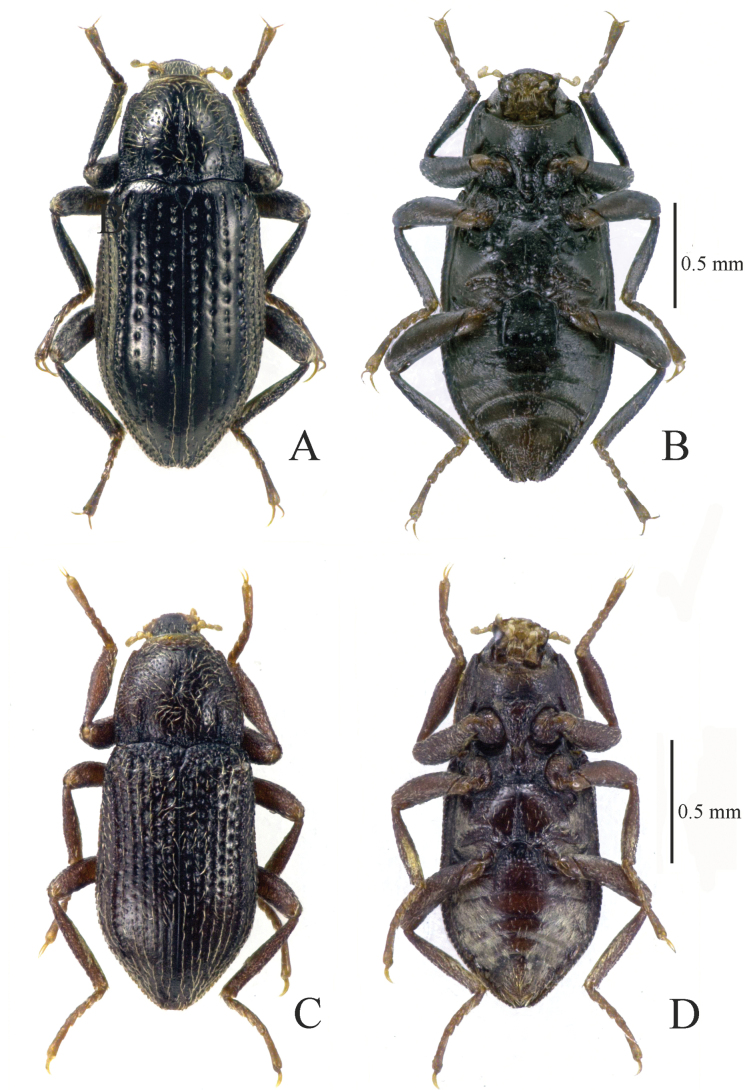
Habitus. **A, B.***Zaitzevia disparilis* sp. nov.; holotype, male; **C, D.***Z. hybrida* sp. nov., holotype, male; **A, C.** Dorsal view; **B, D.** Ventral view.

##### Description.

**Male** (holotype). Body broadly obovate. BL 2.6 mm, BW 1.2 mm. Antennae yellowish brown, dorsum black, ventral side brown to black, legs reddish brown to dark brown except tarsi light brown.

Labrum microreticulate in basal 0.1, distal 0.9 smooth and shiny, sparsely punctate and pubescent. Anterior margin broadly rounded, not emarginate, densely pubescent laterally. Clypeus coarse, densely pubescent, sparsely granulate. Frons densely pubescent, with a few small granules near inner sides of eyes.

Pronotum (PL 0.7 mm, PW 0.9 mm) broadest at base, gradually narrowing anteriad. Lateral margin slightly rimmed. Anterior corners acute, slightly produced, posterior corners right-angled. Disc smooth and shiny, densely punctate, and sparsely pubescent. Area near anterior and posterior corners and in front of scutellum coarse, finely granulate. Median sulcus reaching from basal 0.2–0.5, narrow, not broadened medially. Sublateral carinae extending from base to basal 0.4, slightly raised.

Elytra (EL 1.9 mm, EW 1.2 mm) broadest at distal 0.33, slightly narrowed anteriorly and distinctly attenuate posteriorly. Strial punctures moderately large, especially in the middle area, separated by ~ 0.5–1.0 × their diameters, becoming smaller and shallower towards declivity, separated by 2–3 × their diameters. Intervals flat, more or less smooth and glabrous, hardly punctate, and hardly pubescent. Intervals 5, 7 and 8 carinate; carinae on interval 5 extending from basal 0.2 to apex; carinae on intervals 7 and 8 extending from basal 0.1 to apex. Elytral apices densely granulate, separately rounded (in posterior view).

Prosternum laterally densely pubescent; prosternal process gradually narrowed from base to distal 0.2, distinctly narrowed in distal 0.2, apex narrowly rounded, lateral margin distinctly rimmed; disc coarse with some large granules, sparsely punctate. Disc of metaventrite broadly and distinctly impressed, smooth and shiny, with a few very small punctures and large granules; with a row of large punctures behind of mesocoxa and another row of small punctures in front of metacoxa; median sulcus complete, gradually narrowing anteriad; lateral margin of disc in the median part (between meso- and metacoxae) with a large hump covered with a few distinct granules; lateral areas densely pubescent and sparsely granulate.

Ventrite I moderately densely punctate, with a pair of admedian carinae; disc more or less impressed, anterior margin raised. Middle of ventrites II–IV smooth and shiny, sparsely and finely punctate. Lateral areas of ventrites I–V densely pubescent. Ventrite V densely pubescent and sparsely granulate; lateral margins V fringed with short spines; apical margin weakly emarginate medially; lateral corners not forming distinct teeth.

Protibiae slightly enlarged.

**Aedeagus**. 1.2 mm long, elongate, cylindrical. Penis ~ 1.9 × as long as phallobase, gradually broadened in basal 0.33, then almost subparallel to basal 0.67, distinctly acuminate in distal 0.33, apex acute. Endophallus with characteristic sclerotizations resembling a crown. Parameres very slim, reaching apical 0.2 of penis.

**Females**. Metaventrite not distinctly impressed; pair of humps between meso- and metacoxae smaller, without granules. Disc of ventrite I glabrous, very sparsely punctate, partly and faintly microreticulate, concave in posterior 0.5. Apex of ventrite V straight or weakly rounded, not emarginate. Protibiae not enlarged.

##### Measurements.

Males: BL 2.50–2.65 mm (*n* = 10), BW 1.1–1.2 mm (*n* = 6); females: BL 2.3–2.7 mm (*n* = 10), BW 1.1–1.2 mm (*n* = 5).

##### Distribution.

Hubei, Shaanxi.

##### Etymology.

The epithet is derived from the Latin noun *coronifer* (carrier of a crown) and refers to the crown-like sclerotizations of the endophallus.

#### ﻿ Zaitzevia disparilis sp. nov.

Taxon classificationAnimaliaColeopteraElmidae

5B246B76-E032-5B2E-A681-F05C9119A4AC

https://zoobank.org/E4401A5A-0006-4D5D-A7FE-647ABD8E63F2

Figs 3A, B, 8A–C, 12A–C

##### Material examined.

(822 exs) ***Holotype***: China • ♂ (IAECAS): “China: Shaanxi, \ Foping County \ Gaozhuanggou | 33°34′18″N, 107°57′56″E \ 1035 m, 2019. VI. 21 \ Leg. Tong”. ***Paratypes***: China, Shaanxi • 6 ♂♂, 3 ♀♀, 32 exs (IAECAS), the same data as holotype • 3 exs (NMW): “China: Shaanxi, 1.6.1998 \ 130 km SE Baoji, ca. 1 100 m \ Taibai Shan Forest Park \ leg. M. Wang (CWBS 307)” • 10 exs (NMW): “China: Shaanxi, 7.6.1998 \ 7 km SE Liuba, ca. 1300m \ Taoyuanpu Village env. \ leg. M. Wang (CWBS 316)” • 1 ♀ (CPE): “China: Shaanxi, Qin Ling Shan \ 108.49 E,33.55N [coordinates obviously unprecise], River Valley \ 40 km S Xian, Autoroute km 50 \ River bank, 1200 m \ 31.08.1995, leg. A.Pütz” • 11 exs (CBC, NMW): “China, 17. – 22.VI. \ Shaanxi prov. 1991 \ Hua Shan peak env. \ 100 km E of Xi’an \ Z. Kejval lgt.”.

##### Additional material.

China, Gansu • 1 ♀ (NMW): “China: Gansu, 15.6.1998 \ Wen Co., Bikou env. \ Dong Gou River, ca. 950m \ leg. M. Wang (CWBS 326)”; Anhui • 437 exs (NMW): “China: Anhui, Dabie Shan \ 40km N Yuexi, 5.11.1997 \ env. Gui Xing Di, 800m \ leg. Schönmann [respectively: “leg. M. Wang”] (CWBS 295)” • 220 exs (NMW): “China: Anhui, Dabie Shan \ 20km N Yuexi, 6.11.1997 \ env. Shi Guan, 900m \ leg. Schönmann [respectively: “leg. M. Wang”] (CWBS 296)” • 27 exs (NMW): “China: Anhui, Dabie Shan \ 20km N Yuexi, 6.11.1997 \ env. Shi Guan, 950–1000m \ leg. Schönmann [respectively: “leg. M. Wang”] (CWBS 297)” • 82 exs (NMW): “China: Anhui, Dabie Shan \ 50km NW Yuexi, 7.11.1997 \ Huang Liyan/Baojia, 1050m \ leg. Schönmann [respectively: “leg. M. Wang”] (CWBS 298)” • 8 exs (NMW): “China: Anhui, Dabie Shan \ 50km NW Yuexi, 8.11.1997 \ Huang Liyan/Baojia, 1050m \ leg. Schönmann [respectively: “leg. M. Wang”] (CWBS 299)” • 17 exs (NMW): “China: Anhui, Dabie Shan \ 25km N Yuexi, 9.11.1997 \ env. Shi Guan, 1000m \ leg. Schönmann [respectively: “leg. M. Wang”] (CWBS 301)”; Hunan • 16 exs (NMW): “China, NW-Hunan 1993 \ Wulingyuan, N Dayong \ Zangjiajie [Zhangjiajie], 30. 10.,450m \ leg. Schönmann (4) [CWBS 23]” • 12 exs (NMW): “China, NW-Hunan 1993 \ Wulingyuan, N Dayong \ Suoxiyu, 31. 10., 400m \ leg. Schönmann (5) [CWBS 24]”; Guizhou • 1 ♀ (NMW): “China: Guizhou, Zunyi Pref. \ 25km NE Zunyi City \ ca. 1000m, 3.8.1997 \ leg. M. Wang (CWBS 278)” • 1 ♀ (NMW): “China: Guizhou, Leishan Co. \ SE Kaili, NE Leishan \ Leigong Shan, E - slope \ 26°26.11'N, 108°16.08'E \ Wunang River \ 13.6.2001, ca. 9-00 m [sic] \ leg. Schillhammer & Wang (CWBS 432)”.

##### Differential diagnosis.

*Zaitzevia disparilis* sp. nov. is a rather small species (BL 1.46–2.00 mm), characterized by the often remarkably unequal size of the elytral punctures, by the elytral carinae being located on intervals 5–7, and the conspicuous sclerotizations of the endophallus.

In habitus and body size it somewhat resembles *Z. tsushimana* from Jilin, which is on average longer (BL 1.8–2.2 mm) and differs, among other characters, in the longer median pronotal sulcus (see Jäch and Boukal 1995: fig. 59 [as “*Zaitzevia* sp. (Jilin)”]; Iwata et al. 2022: fig. 3G), the flat elytral intervals (only rarely flat in *Z. disparilis* sp. nov.) and the structure of the endophallic sclerotizations.

##### Description.

**Male** (holotype). BL 1.8 mm, BW 0.8 mm. Body elongate obovate. Dorsum and ventral side dark brown, antennae yellowish brown. Legs dark brown but tarsi reddish brown. Labrum wider than long. Basal 0.33 microreticulate, distal 0.67 densely punctate and pubescent, with fringe of long setae laterally. Clypeus and frons densely pubescent and sparsely granulate.

Pronotum (PL 0.55 mm, PW 0.6 mm) broadest at base, gradually narrowing anteriad, lateral margin narrowly rimmed. Anterior corners acute, slightly produced, posterior corners rectangular. Disc smooth and shiny, punctures sparser than on lateral parts. Areas near anterior and posterior corners coarse, densely and finely granulate. Median groove moderately wide, extending from basal 0.2–0.6. Lateral carinae present in basal 0.4.

Elytra (EL 1.25 mm, EW 0.8 mm) subparallel in basal 0.67, distal 0.33 distinctly attenuate. Strial punctures in basal 0.5 unequal in size and depth, separated by 1.0–1.5 × their diameters, being deepest in the third stria, becoming smaller and shallower on declivity (separated by 2–3 × their diameters); striae not reaching elytral apex. Elytral intervals weakly convex, smooth and shiny, with only a few small setal punctures; interval 4 very slightly more convex than intervals 1–3; intervals 5–7 carinate (due to fusion of elytral striae 5 and 6), carina on interval 5 extending from basal 0.1 to apex, carinae on intervals 6 and 7 complete, merging at basal ~ 0.1. Elytral apices narrowly conjointly rounded.

Prosternal disc coarse, sparsely punctate and pubescent, lateral areas densely tomentose. Prosternal process gradually narrowed in basal 0.8, then distinctly narrowed in distal 0.2, apex narrowly rounded. Disc of prosternal process sparsely punctate and pubescent. Metaventrite shiny, with a few larger elongate punctures, slightly impressed medially; median sulcus present in basal 0.7, distinctly narrowed from base to apex. Lateral areas densely pubescent. Posterior margin of metaventrite impressed, with two large impressions behind of mesocoxae, and a row of middle-sized punctures in front of metacoxae.

Disc of ventrite I flat, smooth, and shiny, with only a few punctures, and a transverse row of few granules posteriorly; anteriorly impressed and wrinkled; laterally bordered by strong admedian carinae. Middle of ventrites II–IV and basal 0.33 of ventrite V smooth and shiny, with only a few punctures. Distal 0.67 of ventrite V densely pubescent and granulate. Lateral areas of ventrites I–IV densely pubescent. Lateral margins of ventrite V fringed with short spines; apex subtruncate, lateral apical corners covered by a brush of stiff setae.

**Aedeagus**. 0.8 mm long, elongated, and cylindrical. Penis ~ 2.2 × as long as phallobase. Penis gradually broadened in basal 0.25, then almost subparallel to apical 0.25, then distinctly narrowed toward cuspidal apex. Sclerotizations of endophallus in median part of penis (basal 0.3–0.7) resembling a “bowknot” (in ventral/dorsal view), consisting of a pair of ovoid structures, each with an apical curved tooth and a moderately long, thin basal rod. Parameres short, clinging to penis, reaching apical 0.45 of penis.

**Females**. Disc of metaventrite more evenly convex or evenly flattened than in males, without larger punctures or granules; ventrite I less impressed anteriorly, without distinct wrinkles; ventrite V very similar to male, but more sparsely granulate; apex subtruncate or weakly rounded.

##### Measurements.

(type specimens) Males: BL 1.68–1.90 mm (*n* = 10), BW 0.75–0.80 mm (*n* = 7); females: BL 1.80–1.96 mm (*n* = 10), BW 0.75–0.80 mm (*n* = 3).

Including the additional material, the BL of *Z. disparilis* is 1.46–2.00 mm. The smallest male (Anhui, CWBS 299) is 1.50 mm long, the smallest female (Anhui, CWBS 301) 1.46 mm, and the largest female (Hunan, CWBS 24) 2.00 mm.

##### Distribution.

Anhui, Gansu, Guizhou, Hunan, Shaanxi. Remarkably, there are no records from Hubei at present.

##### Etymology.

The name is derived from the Latin adjective *disparilis* (disparate, different, unequal), referring to the differently sized and variably deep impressed elytral punctures.

##### Remarks.

We think that all specimens recorded from these five provinces belong to the same species, but due to the morphological variability (e.g., body length, size and depth of elytral punctures, granules of elytral intervals sometimes reaching elytral base) we do not designate the specimens from Anhui, Gansu, Guizhou, and Hunan as paratypes. Confirmation of the conspecific molecular data would be highly desired, especially in case of the material from Gansu and Guizhou from where only females are currently known.

#### ﻿ Zaitzevia hybrida sp. nov.

Taxon classificationAnimaliaColeopteraElmidae

1015FD05-253F-54C6-8A74-BD25083B13C0

https://zoobank.org/CEDF5847-9F1A-4EBA-A4C0-B2EF0F70DA16

Figs 3C, D, 9A–C, 12D–F

##### Material examined.

(45 exs) ***Holotype***: China • ♂ (IAECAS): “China, Shaanxi, Qingling \ Ningshan County | Yaowangtang, 1286 m \ 2005.6.10, Wangm [leg. Wang Miao]”. ***Paratypes***: China, Shaanxi • 4 ♂♂, 3 ♀♀ (IAECAS): “China-Shaanxi \ Zhouzhi County I. [leg.] W [Wang] | Houzhenzi ca. 1200 m \ 2.VI. 1998” [CWBS 308] • 8 ♂♂, 8 ♀♀ (NMW): “China: Shaanxi, 2.6.1998 \ Zhouzhi County, ca. 1200m \ 2km W Houzhenzi Nat. Res. \ leg. M. Wang (CWBS 308)” • 1 ♀ (NMW): “China: Shaanxi, 4.6.1998 \ Foping County, ca. 1 300m \ 5km S Longcaoping \ leg. Wang (CWBS 312)” • 2 ♂♂, 6 ♀♀ (NMW): “China: Shaanxi, 6.6.1998 \ Ningshan Co., ca. 1 500m \ 10 km NE Xunyangba \ leg. M. Wang (CWBS 315)” • 1 ♂ (NMW): “China, 17. – 22.VI. \ Shaanxi prov. 1991 \ Hue Shan peak env. \ 100 km E of Xi’an \ Z. Kejval lgt.”.

##### Additional material.

China, Gansu • 2 ♂♂ (NMW): “China: Gansu, 13.6.1998 \ Wen Co., ca. 1100m \ 4km N Shangdan Village \ leg. M. Wang (CWBS 322)” • 1 ♀ (NMW): “China: Gansu, 14.6.1998 \ Wen Co., ca. 1 150m \ 1km N Shangdan Village \ leg. M. Wang (CWBS 323)”; Sichuan • 2 ♂♂ (NMW): “China: Sichuan, 13.6.1996 \ W Ya’an, 20 km W Tianquan \ Dayuxi stream, 1200m \ leg. Ji & Wang (CWBS 235)” • 1 ♀ (NMW): “China: Sichuan, 29.7.1998 \ Mao Xian Co., Jiuding Shan \ 10 km NE Mao Xian, ca. 1950m \ Schönmann, Ji, Wang (CWBS 336)” • 1 ♀ (NMW): “China: Sichuan, 30.7.1998 \ Mao Xian Co., Jiuding Shan \ 20 km NE Mao Xian, ca. 1650m \ Schönmann, Ji, Wang (CWBS 337)” • 1 ♀ (NMW): “China: Sichuan, 30.7.1998 \ Mao Xian Co., Jiuding Shan \ 15 km NE Mao Xian, ca. 1950m \ Schönmann, Ji, Wang (CWBS 338)” • 1 ♀ (NMW): “China: Sichuan, 30.7.1998 \ Mao Xian Cty., Jiuding Shan \ 6 km NE Mao Xian, ca. 1750m \ Schönmann, Ji, Wang (CWBS 340)”; Yunnan • 1 ♂ (NMW): “China-Yunnan 24.–29.6. \ 50 km N Lijiang, 1993 \ Yulongshan Nat. Res. \ E.Jendek & O.Sausa leg.” • 3 ♂♂, 6 ♀♀ (NMW): “China-Yunnan 14.–21.6. \ 100 km W Baoshan, 1993 \ Gaoligongshan Nat. Res. \ E.Jendek & O.Sausa leg.”.

##### Differential diagnosis.

*Zaitzevia hybrida* sp. nov. is a small species (BL 1.50–1.86 mm), which differs significantly from all other species described in the genus *Zaitzevia* in possessing characters that are typically found in *Zaitzeviaria* Nomura, 1959, i.e., elytra with only two instead of three carinae, and elytral the plastron being confined to the area between the lateral margin and interval 7 (therefore not reaching interval 5). This species is furthermore characterized by the enlarged male protibiae.

##### Description.

**Male** (holotype). BL 1.7 mm, BW 0.7 mm. Dorsum dark brown, ventral side and legs reddish brown except tarsi. Antennae, mouth parts, and tarsi yellowish brown.

Labrum wider than long, anterior margin not emarginate, basal 0.5 microreticulate, distal 0.5 smooth and shiny, sparsely punctate and pubescent laterally. Clypeus and frons densely punctate and pubescent.

Pronotum (PL 0.5 mm, PW 0.6 mm) broadest at basal 0.3, slightly narrowed posteriorly and distinctly attenuate anteriorly, posterior corners rectangular. Disc smooth and shiny, densely pubescent shallowly punctate. Areas near anterior and posterior corners coarsely granulate. Median groove short and shallowly impressed, extending from basal 0.4–0.7. Lateral carinae present in basal 0.4, joined by a distinct mesial groove. Base of pronotum with a group of granules medially.

Elytra (EL 1.2 mm, EW 0.75 mm) broadest at basal 0.67, slightly narrowed anteriorly and distinctly attenuate posteriorly, apices narrowly conjointly rounded. Strial punctures large in basal 0.5, separated by 0.5–1.0 × their diameters; punctures becoming smaller and well separated in distal 0.5. Intervals smooth and shiny, each with a row of setae; intervals 7 and 8 distinctly carinate, extending from base to declivity. Base of elytra narrowly granulate. Lateral margins distinctly denticulate.

Prosternum coarsely punctate and pubescent. Prosternal process distinctly narrowed from base to narrowly rounded apex; lateral margin distinctly rimmed. Disc convex, coarse, sparsely punctate and pubescent. Metaventrite slightly and broadly impressed, disc smooth and shiny, sparsely punctate and pubescent; median groove extending from base to anterior 0.1, gradually narrowed towards apex; lateral area densely pubescent and sparsely granulate; each side with two large elongate pits behind mesocoxae and a row of large punctures is in front of metacoxae.

Disc of ventrite I convex, largely smooth and shiny, sparsely punctate; along anterior margin slightly impressed with densely arranged, deeply impressed grooves; laterally bordered by strong admedian carinae. Discs of ventrites II–IV and basal 0.2 of ventrite V smooth and shiny; sparsely punctate and pubescent. Lateral sides of ventrites I–IV densely pubescent and sparsely granulate. Apex of ventrite V subtruncate, lateral apical corners rounded, bearing some stiff setae. Protibia distinctly enlarged.

**Aedeagus**. 0.69 mm long, elongate. Penis ~ 3.5 × as long as phallobase, very slightly asymmetrical, subparallel in basal 0.4, then gradually narrowed; apex subacute. Sclerotizations of endophallus in basal 0.5 of penis, consisting of a pair of elongate ovoid structures (in basal 0.3–0.5) and a pair of long thin contiguous basal rods. Parameres short, reaching apical 0.33 of penis.

**Females**. Protibiae not distinctly enlarged. Sexual dimorphism of metaventrite and abdominal ventrites poorly developed; ventrite I anteriorly less strongly impressed and less distinctly wrinkled; apex of ventrite V variable in both sexes: weakly emarginate, subtruncate or weakly rounded.

##### Measurements.

Males: BL 1.50–1.86 mm (*n* = 18), BW 0.75–0.80 mm (*n* = 6); females: BL 1.68–1.86 mm (*n* = 17), BW 0.75–0.80 mm (*n* = 3).

##### Distribution.

Gansu, Shaanxi, Sichuan, Yunnan, ? Himalaya.

Comparison with Himalayan specimens (Himachal Pradesh, Nepal, Bhutan) indicates that *Zaitzevia hybrida* seems to occur there as well. However, it will be necessary to confirm this assumption by molecular data.

##### Variability.

Due to the morphological variability of the material examined (e.g., protibiae, elytral intervals, aedeagi), we do not designate the specimens from Gansu, Sichuan, and Yunnan as paratypes. Examination by molecular methods would be highly desired. The single male from Yulongshan (Yunnan) is 1.68 mm long and its aedeagus measures 0.69 mm; its external morphology agrees quite well with specimens of *Z. hybrida* from Gansu, Shaanxi, and Sichuan. However, the three males from Gaoligongshan (Yunnan) are 1.46–1.64 mm long, and their aedeagi are slimmer and shorter (0.61–0.63 mm) than in the males from the other populations (0.68–0.70 mm). Furthermore, they differ externally in the distinctly less densely punctate elytral striae and the flatter elytral declivity; genetically, they differ in the ovoid sclerotization of the endophallus being distinctly smaller. Therefore, these nine specimens from Gaoligongshan (Yunnan) listed above may well represent a different species, which should be confirmed by DNA sequencing.

The enlargement of the male protibiae is obviously allometric. In larger specimens the tibiae are disproportionately larger than in smaller ones.

As in other species of the genus *Zaitzevia*, the size and number of the elytral punctures, and especially the width and convexity of the elytral intervals 2–6 are quite variable; these intervals can be flat, moderately convex or carinate, at least in the anterior 0.33. In some specimens, the elytral apices are separately rounded.

The shape of the aedeagus of *Z. hybrida* is normally symmetrical; in the holotype it is hardly noticeably asymmetrical, and in the two males from Gansu (CWBS 322), one is very slightly asymmetrical and the other one even more distinctly so.

##### Etymology.

The epithet is derived from the Latin noun *hybrida* (hybrid) referring to the fact that *Zaitzevia hybrida* and a number of other undescribed species from China and the Himalaya combine diagnostic characters of *Zaitzevia* and *Zaitzeviaria*.

##### Remarks.

At a first glance, *Zaitzevia hybrida* seems to belong to the genus *Zaitzeviaria*, based on the small size and the elytral carinae being located on intervals 7 and 8. On the other hand, it shares a number of characters with *Zaitzevia*. Therefore, and in the absence of any molecular data, we have decided to place this species tentatively in the genus *Zaitzevia*. For further information, see the Discussion.

#### ﻿ Zaitzevia cf. chenzhitengi

Taxon classificationAnimaliaColeopteraElmidae

Jiang & Wang, 2020

F71BA7B7-1D29-5530-8096-72D840A5AD95

Figs 4A, B, 10D–F

Zaitzevia chenzhitengi Jiang and Wang 2020: 232; Bian and Zhang 2022: 265; Jiang and Chen 2023: 204 (key).

##### Material examined.

China, Shaanxi • 1 ♂ (IAECAS): “China: Shaanxi, \ Qinling Northwest \ A & F University forestry center | Huoditang \ 1974 m, 2005.6.12, Leg. Wang M.”; Sichuan • 2 ♂♂, 2 ♀♀ (NMW): “China: Sichuan \ Emeishan \ 160km SSW Chengdu \ 1530m (4a) [CWBS 52] \ 22.6.1994 \ leg. Schillhammer”; Yunnan • 1 ♂ (IAECAS): “China: Yunnan (20) \ Nujiang, \ Lushui, Pianma | 26°0'3"N, 98°39'42"E \ 2392m, 2018.10.25. \ Leg Peng, Zhu, Dong”; 3 ♂♂, 3 ♀♀ (IAECAS): “CHINA: Yunnan (25) \ Nujiang, Lushui \ Pianma | 26°0'26"N, 98°38'54"E \ 2172 m, 2019.7.6 \ Leg.Peng, Zhu, Dong” • 2 ♂♂, 4 ♀♀ (NMW): “China NW-Yunnan \ 15km N Lijiang \ 2800m, 6.7.1994 \ leg. JI (16) [CWBS 60]”.

##### Taxonomy.

**Figure 4.**
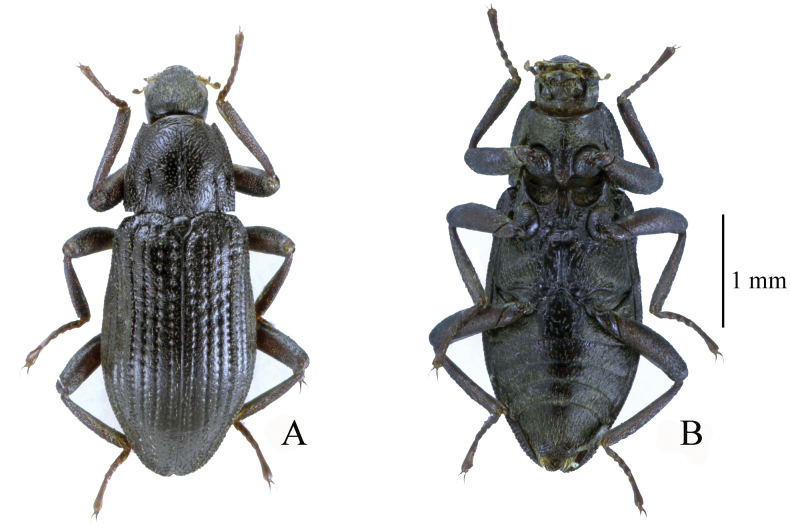
Habitus. **A, B.**Zaitzevia cf. chenzhitengi Jiang & Wang, 2020, male from Shaanxi. **A.** Dorsal view; **B.** Ventral view.

*Zaitzevia chenzhitengi* was described by Jiang and Wang (2020) based on one male and one female from northern Sichuan. Two years later, Bian and Zhang (2022) newly recorded this species from Shaanxi and Yunnan and published photographs of the male habitus and the aedeagus of a specimen from Shaanxi. However, after having examined additional material, which we compared with the original description, we want to express our doubts about the identity of the specimens listed above. Although not explicitly mentioned in the written description by Jiang and Wang (2020), the elytral apices of the holotype are distinctly produced and conjointly acuminate in the figures (see Jiang and Wang 2020: figs 1A, 2F), while none of the nine males from the three provinces examined by us has distinctly acuminate elytral apices (see Fig. 4A). Furthermore, the body form of the holotype of *Z. chenzhitengi* is clearly more slender and parallel-sided, and the anterior angles of the pronotum seem to be less acute. Despite the external differences, the aedeagi are remarkably similar, but the endophallic teeth appear to be smaller and situated further proximally in the holotype of *Z. chenzhitengi*. More material from the type area of *Z. chenzhitengi* and/or DNA sequencing will be necessary to clarify the identity of “Z. cf. chenzhitengi”.

##### Distribution.

Shaanxi, Sichuan, Yunnan.

### ﻿Key to the *Zaitzevia* species of Shaanxi

**Table:** 

1	Body large, BL > 3.0 mm	**2**
–	Body smaller, BL < 3.0 mm	**3**
2	Disc of pronotum rugose in basal 0.3 (Figs 1A, C, 5A), apex of penis (Fig. 5A, B) broadly rounded, distinctly curved dorsad in lateral view	***Z. robusta* sp. nov.**
–	Disc of pronotum smooth and shiny in basal 0.3 (Fig. 4A), apex of penis (Fig. 10D, E) characteristically arrowhead-shaped, not or very weakly curved dorsad in lateral view	**Z. cf. chenzhitengi**
3	BL < 2.0 mm, elytral carinae on intervals 5–7 or 7 and 8	**4**
–	BL > 2.0 mm, elytral intervals 5, 7 and 8 carinate	**5**
4	Elytral intervals 7 and 8 carinate, male protibiae inflated (Figs 3C, D, 9A, B); endophallic sclerotizations restricted to basal 0.5 (Fig. 12D–F)	***Z. hybrida* sp. nov.**
–	Elytral intervals 5–7 carinate, male protibiae not inflated (Fig. 3A, B); endophallic sclerotizations restricted to basal 0.3–0.7, resembling a “bowknot” in ventral/dorsal view (Fig. 12A, B)	***Z. disparilis* sp. nov.**
5	Habitus as in Fig. 2A; pilosity of dorsum distinctly pronounced (if not rubbed off, as in many older specimens); granules on elytral interval 5 reaching elytral base, shoulders therefore not glabrous; aedeagus (Fig. 11A–C) apically subacute but not distinctly produced, endophallus with a pair of distinct distal teeth	***Z. pilosa* sp. nov.**
–	Habitus as in Fig. 2C; pilosity of dorsum sparse; granules on elytral interval 5 lacking in basal 0.2 of elytra, shoulders therefore glabrous; aedeagus (Fig. 11D–F) apically distinctly produced, endophallus without teeth	***Z. coronifer* sp. nov.**

**Figure 5.**
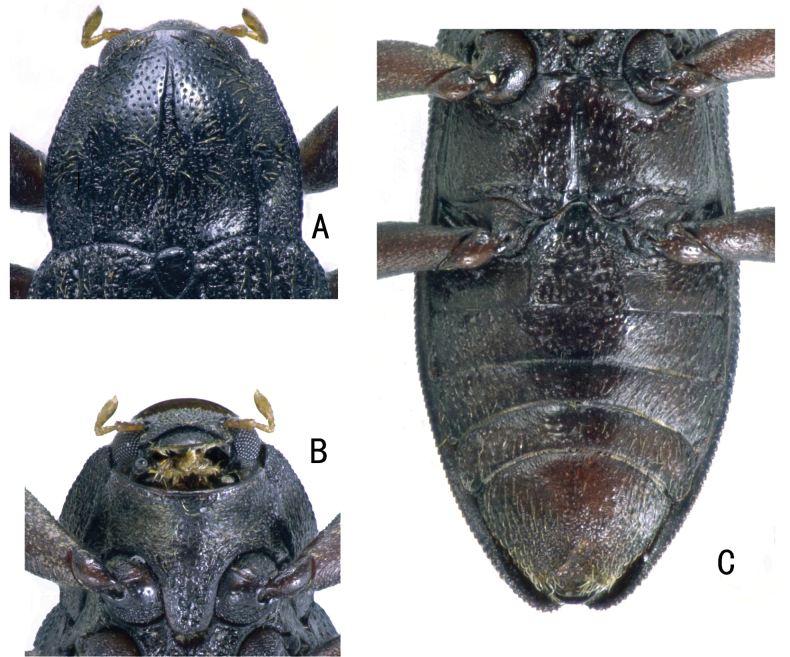
*Zaitzevia robusta* sp. nov., holotype, male; **A.** Pronotum; **B.** Prosternum; **C.** Metaventrite and abdominal ventrites.

**Figure 6.**
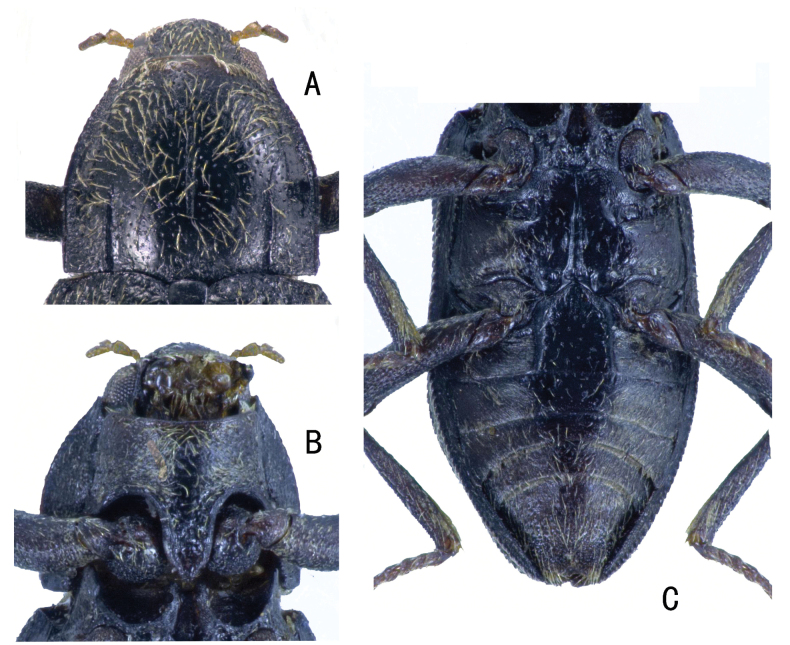
*Zaitzevia pilosa* sp. nov., holotype, male; **A.** Pronotum; **B.** Prosternum; **C.** Metaventrite and abdominal ventrites.

**Figure 7.**
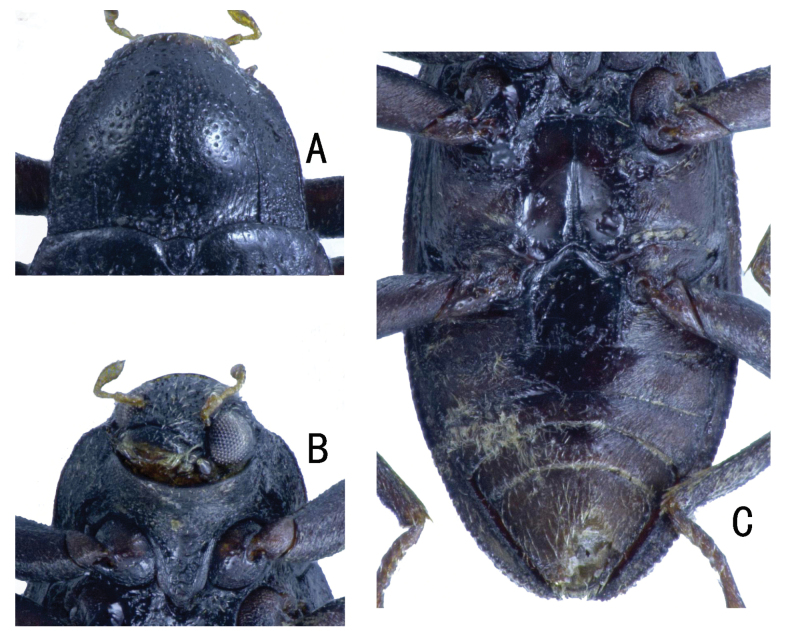
*Zaitzevia coronifer* sp. nov., holotype, male; **A.** Pronotum; **B.** Prosternum; **C.** Metaventrite and abdominal ventrites.

**Figure 8.**
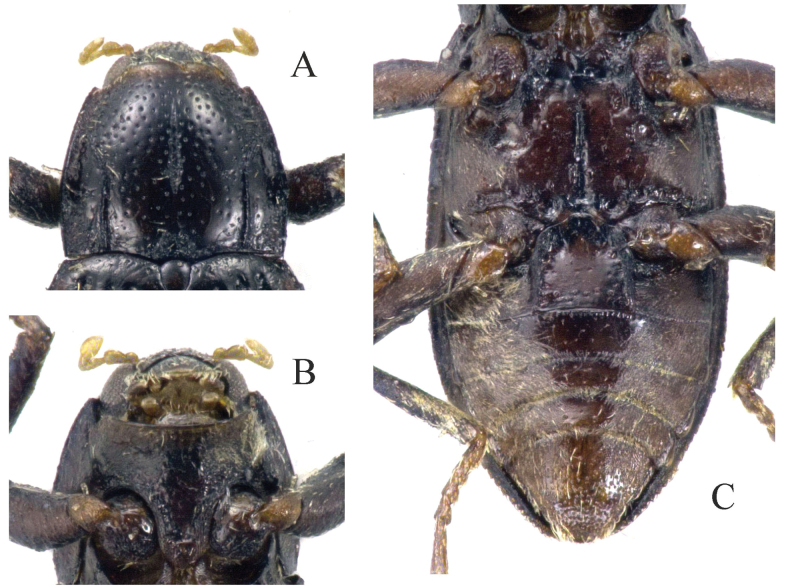
*Zaitzevia disparilis* sp. nov., holotype, male; **A.** Pronotum; **B.** Prosternum; **C.** Metaventrite and abdominal ventrites.

**Figure 9.**
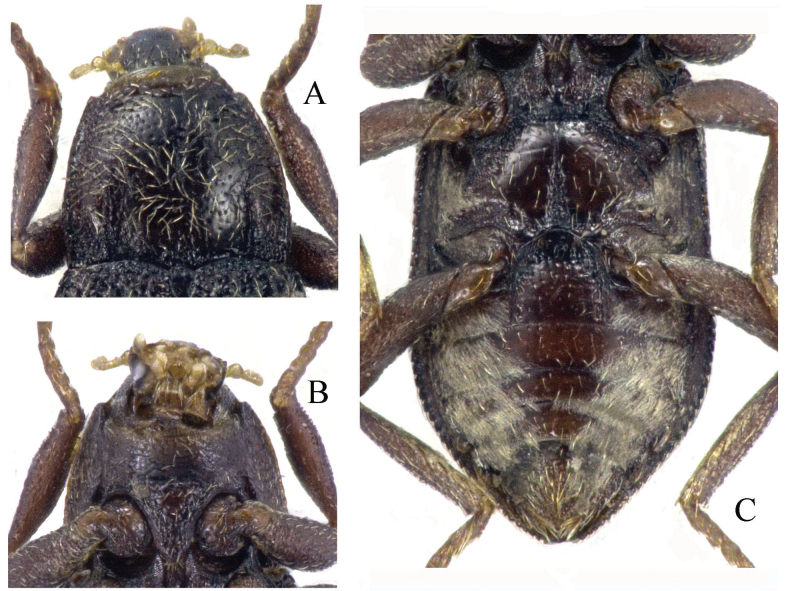
*Zaitzevia hybrida* sp. nov., holotype, male; **A.** Pronotum; **B.** Prosternum; **C.** Metaventrite and abdominal ventrites.

## ﻿Discussion

The genus *Zaitzevia* is very common in China. Although 21 species are now already described from this country (including the ones added herein), there are still dozens of undescribed species from many CWBS localities housed in the NMW. Therefore, we expect that the total number of *Zaitzevia* species actually existing in China is much higher than 50. Apart from Chinese specimens, the NMW houses thousands of unidentified Asian specimens of Macronychini. Many of these can not be attributed to existing genera based on their morphology, either because they belong to undescribed genera or, as in most cases, because they possess character sets that do not allow unambiguous generic placement (e.g., in *Aulacosolus* Jäch & Boukal, 1997 or *Indosolus* Bollow, 1940; in *Eonychius* Jäch & Boukal, 1996 or *Homalosolus* Jäch & Kodada, 1996; in *Rhopalonychus* Jäch & Kodada, 1996 or *Zaitzeviaria*). Undoubtedly, *Zaitzevia hybrida* is such a species that could be placed in two different genera, because it possesses typical characters of both *Zaitzevia* and *Zaitzeviaria*. At a first glance, *Zaitzevia hybrida* seems to belong to the genus *Zaitzeviaria*, based on the size and the elytral carinae being located on intervals 7 and 8. However, in several other characters, this species is seemingly closer to the type species of *Zaitzevia*, i.e., *Zaitzevia solidicornis*, than to *Zaitzeviaria brevis* (Nomura, 1958), the type species of *Zaitzeviaria*. For instance, the median pronotal groove reaches the pronotal base in typical *Zaitzeviaria*, while it does only very exceptionally so in *Zaitzevia* (e.g., in *Z. tsushimana*). The pronotal sublateral furrows are very faint in *Zaitzeviaria*, but usually very deeply impressed in *Zaitzevia* (except in a few species, e.g., *Z. tangliangi*). In *Zaitzeviaria*, the scutellum is usually more rounded, while it is more elongate in *Zaitzevia*. In cross section, the elytra are always very convex and strongly rounded in *Zaitzeviaria*, while they are less strongly rounded and dorsally somewhat flattened in *Zaitzevia*. Enlarged protibiae are known to occur in *Zaitzevia* (e.g., *Z. coronifer*), while they are unknown in typical species of *Zaitzeviaria*. In lateral view, the median lobe of *Zaitzeviaria* is in most cases characteristically curved basally, while it is more or less straight in *Zaitzevia*. Apart from these characters, we decided to place *Zaitzevia hybrida* in the genus *Zaitzevia* also because of the aedeagal similarity with *Z. disparilis*. In both species, the endophallic sclerotizations consist of a pair of ovoid structures with basal rods.

**Figure 10.**
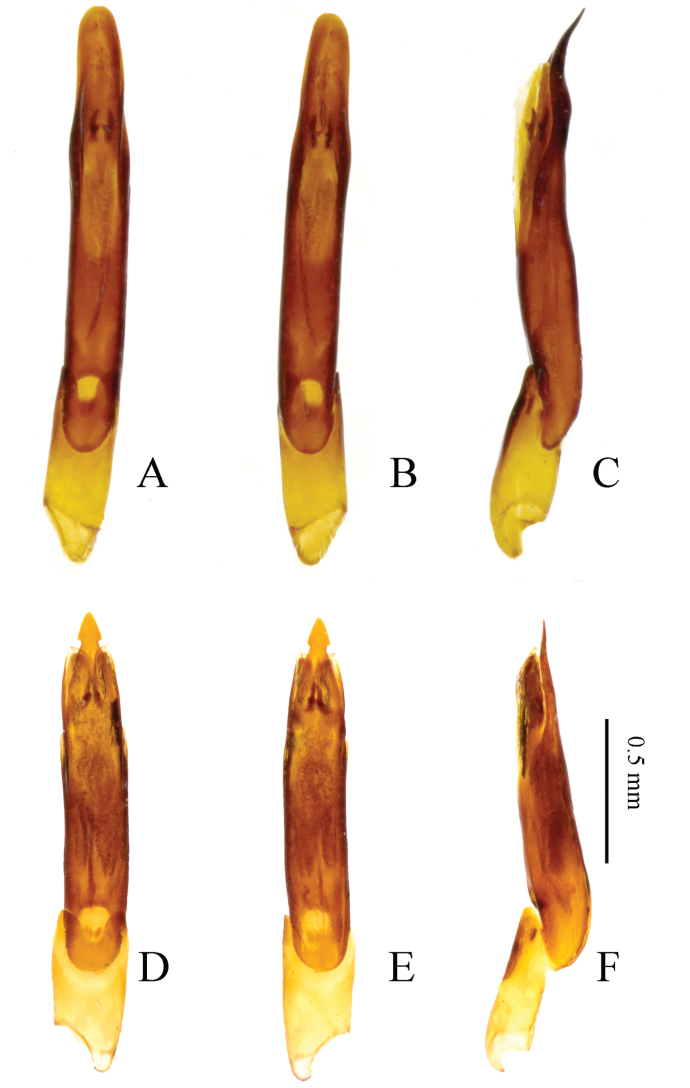
Aedeagus. **A–C.***Zaitzevia robusta* sp. nov., holotype, male; **D–F.**Z. cf. chenzhitengi, specimen from Shaanxi; **A**, **D.** Ventral view; **B**, **E.** Dorsal view; **C**, **F.** Lateral view.

**Figure 11.**
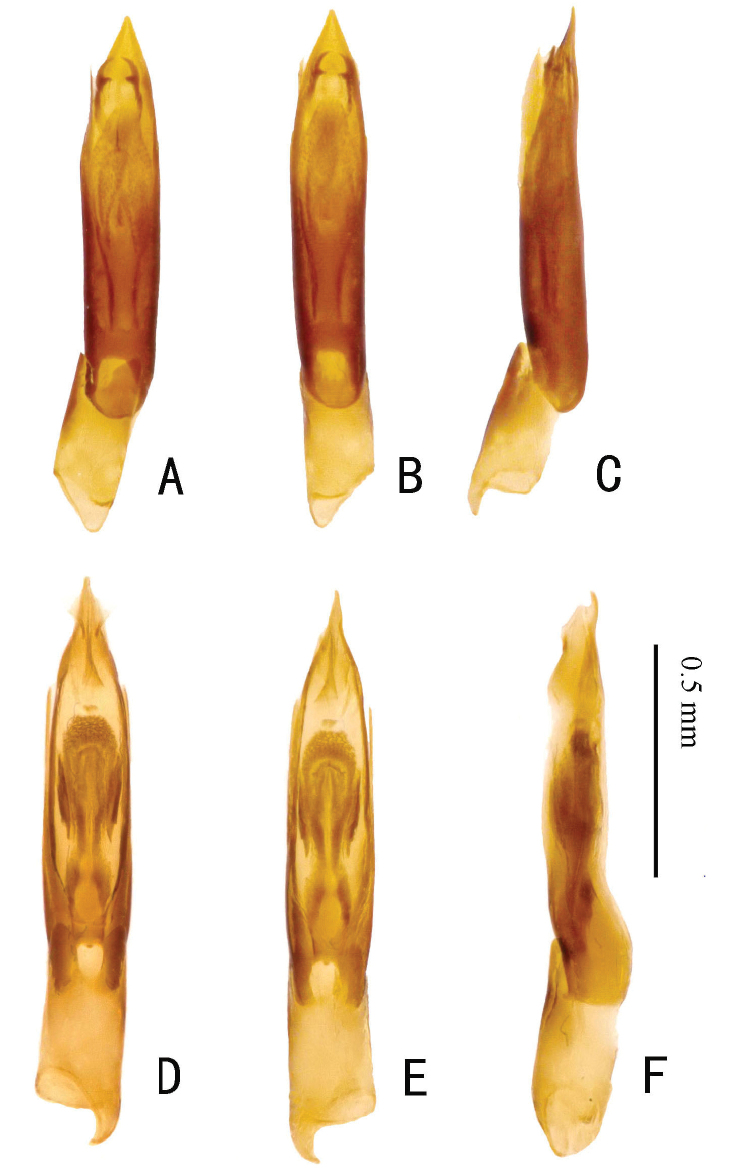
Aedeagus. **A–C.***Zaitzevia pilosa* sp. nov., holotype, male; **D–F.***Z. coronifer* sp. nov., holotype, male; **A, D.** Ventral view; **B, E.** Dorsal view; **C, F.** Lateral view.

**Figure 12.**
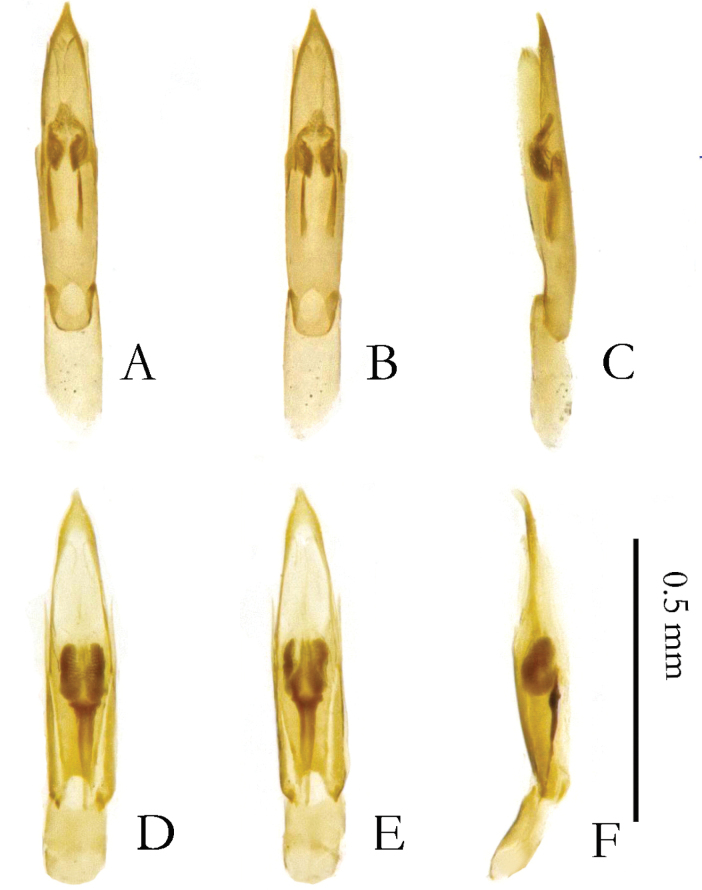
Aedeagus. **A–C.***Zaitzevia disparilis* sp. nov., holotype, male; **D–F.***Z. hybrida* sp. nov., paratype, male; **A, D.** Ventral view; **B, E.** Dorsal view; **C, F.** Lateral view.

*Zaitzevia hybrida* belongs to a group of closely related, partly very similar species that are wide-spread in the Himalaya (Himachal Pradesh, Nepal, Bhutan) and China (Gansu, Hubei, Shaanxi, Sichuan, Tibet, Yunnan). All these species form a monophyletic lineage sharing enlarged male protibiae and a very similar aedeagus (endophallus with large central sclerotisation and long adjoining admedian struts). The most derived species was collected in Hubei (CWBS 539) and has also the profemur (not only the protibia) strongly enlarged. Another group of markedly smaller, more *Zaitzeviaria*-like species, occurs in Myanmar, Thailand, Laos, and Peninsular Malaysia. In one of the Laotian species, the tibia is greatly enlarged and the elytral interval 5 is interestingly slightly raised (although not as ridge-like as in *Zaitzevia*).

The genus *Zaitzevia* is distributed in North America and in Asia, where it occurs from the Russian Far East southwards to Japan and Vietnam and westwards to Myanmar and northwestern India; by far the majority of the species lives in China. The genus *Zaitzeviaria*, as currently defined, is, in contrast, restricted to Asia, where it occurs in a vast area from Sri Lanka northwards to the Himalaya, northeastwards to Japan and southwards to the Philippines, Borneo and Lombok. However, when analyzing the morphological characters of all these species, it becomes evident that *Zaitzeviaria* is in fact a complex of several monophyletic lineages that obviously represent closely related but distinct undescribed genera.

The *Zaitzevia hybrida* species group, as defined above, may actually represent a distinct genus, which is, however, yet to be confirmed by phylogenetic analyses also using molecular data. Since such an analysis is currently not available, we tentatively place this morphologically intermediate group in the genus *Zaitzevia*, mainly because it shares slightly more characters with *Zaitzevia* than with *Zaitzeviaria*.

Furthermore, we want to raise awareness of the currently very unsatisfactory knowledge of the phylogenetic relationships and generic classification of the Macronychini and the difficulty or even impossibility of placing certain species unambiguously in existing genera. Except for the Japanese Macronychini (Kobayashi et al. 2021), no attempt has ever been made to phylogenetically revise the genera of this interesting tribe.

## Supplementary Material

XML Treatment for Macronychini

XML Treatment for Zaitzevia

XML Treatment for Zaitzevia robusta

XML Treatment for Zaitzevia pilosa

XML Treatment for Zaitzevia coronifer

XML Treatment for Zaitzevia disparilis

XML Treatment for Zaitzevia hybrida

XML Treatment for Zaitzevia cf. chenzhitengi

## ﻿Additional information

### Use of AI

No use of AI was reported.

### Author ORCIDs

Dong-Ju Bian https://orcid.org/0000-0002-9324-8026

Manfred A. Jäch https://orcid.org/0000-0002-2990-3604

### Data availability

All of the data that support the findings of this study are available in the main text.

## References

[B1] Bian D-J Hu Y-Q (2024) Two new species of the genus *Zaitzevia* Champion (Coleoptera: Elmidae) from Guangdong Province, China. Zootaxa 5528(1): 118–124. 10.11646/zootaxa.5528.1.10 39646898

[B2] Bian D-J Zhang YI (2022) Three new species of *Zaitzevia* Champion, 1923 from China (Coleoptera: Elmidae: Macronychini). Zootaxa 5190(2): 257–266. 10.11646/zootaxa.5190.2.5 37045170

[B3] Iwata T Hayashi M Yoshitomi H (2022) Revision of the genus *Zaitzevia* (Coleoptera: Elmidae) of Japan. Japanese Journal of Systematic Entomology 28(1): 116–141.

[B4] Jäch MA Boukal DS (1995) Elmidae: 2. Notes on Macronychini, with description of four new genera from China (Coleoptera). In: Jäch MA Ji L (Eds) Water Beetles of China, Vol.I. Zoologisch-Botanische Gesellschaft in Wien and Wiener Coleopterologenverein, Wien, 299–323.

[B5] Jäch MA Ji L (1995) Introduction. In: Jäch MA Ji L (Eds) Water Beetles of China.Vol. I. Zoologisch-Botanische Gesellschaft in Österreich and Wiener Coleopterologenverein, Wien, 5–32.

[B6] Jäch MA Ji L (1998) China Water Beetle Survey (1995–1998). In: Jäch MA Ji L (Eds) Water Beetles of China.Vol. II. Zoologisch-Botanische Gesellschaft in Österreich and Wiener Coleopterologenverein, Wien, 1–23.

[B7] Jäch MA Ji L (2003) China Water Beetle Survey (1999–2001). In: Jäch MA Ji L (Eds) Water Beetles of China.Vol. III. Zoologisch-Botanische Gesellschaft and Wiener Coleopterologenverein, Wien, 1–20.

[B8] Jäch MA Kodada J Brojer M Shepard WD Čiampor F (2016) Coleoptera: Elmidae and Protelmidae. World Catalogue of Insects. Vol. 14. Brill, Leiden, XXI + 318 pp. 10.1163/9789004291775

[B9] Jiang R-X Chen X-S (2023) Three new species of the genus *Zaitzevia* Champion, 1923 (Coleoptera, Elmidae) from China. ZooKeys 1174: 191–206. 10.3897/zookeys.1174.101046 37602199 PMC10436192

[B10] Jiang R-X Wang S (2020) Two new species of the genus *Zaitzevia* Champion, 1923 from China (Coleoptera: Elmidae: Macronychini). Zootaxa 4852(2): 231–238. 10.11646/zootaxa.4852.2.8 33056428

[B11] Jiang R-X Wang S (2021) *Zaitzevia tangliangi* sp. nov. a new riffle beetle from China (Coleoptera: Elmidae: Macronychini). Zootaxa 5061(3): 591–596. 10.11646/zootaxa.5061.3.12 34810605

[B12] Jung SW Jäch MA Bae YJ (2015) : Review of the Korean Elmidae (Coleoptera: Dryopoidea) with descriptions of three new species. Aquatic Insects 36 (2) [2014]: 93–124. 10.1080/01650424.2015.1046457

[B13] Kobayashi T Hayashi M Kamite YT Sota T (2021) Molecular phylogeny of Elmidae (Coleoptera: Byrrhoidea) with a focus on Japanese species: implications for intrafamilial classification. Systematic Entomology 46(4): 870–886. 10.1111/syen.12499

[B14] Nomura S (1958) Notes on the Japanese Dryopoidea (Coleoptera), with two species from Saghalien. Tôhô-Gakuhô 8: 45–59, 2 pls.

[B15] Peng Y-F Bian D-J Wang J-P (2024) *Heterlimnius luyashanensis* sp. n. and *Zaitzevia triangularis* sp. n. from Shanxi Province, China (Coleoptera: Elmidae). Zootaxa 5403(4): 488–494. 10.11646/zootaxa.5403.4.7 38480421

